# Cumulative dispensing of high oral corticosteroid doses for treating asthma in Australia

**DOI:** 10.5694/mja2.50758

**Published:** 2020-09-09

**Authors:** Mark Hew, Vanessa M McDonald, Phil G Bardin, Li Ping Chung, Claude S Farah, Amanda Barnard, Mark S Cooper, Peter G Gibson, John W Upham

**Affiliations:** ^1^ Alfred Hospital Melbourne VIC; ^2^ Centre for Healthy Lungs University of Newcastle Newcastle NSW; ^3^ Monash University Melbourne VIC; ^4^ Fiona Stanley Hospital Perth WA; ^5^ The University of Sydney Sydney NSW; ^6^ Rural Clinical School Australian National University Canberra ACT; ^7^ Concord Hospital Sydney NSW; ^8^ Hunter Medical Research Institute University of Newcastle Newcastle NSW; ^9^ University of Queensland Brisbane QLD

**Keywords:** Asthma, Corticosteroids

## Abstract

**Objective:**

To estimate the level of dispensing of oral corticosteroids (OCS) for managing asthma in Australia, with a particular focus on the cumulative dispensing of doses associated with long term toxicity (≥ 1000 mg prednisolone‐equivalent).

**Design:**

Retrospective cohort study; analysis of 10% random sample of Pharmaceutical Benefits Scheme (PBS) dispensing data.

**Participants, setting:**

People aged 12 years or more treated for asthma during 2014–2018, according to dispensing of controller inhaled corticosteroids (ICS).

**Main outcome measures:**

Number of people dispensed OCS for managing asthma during 2014–2018; proportion who were cumulatively dispensed at least 1000 mg prednisolone‐equivalent. The secondary outcome was the number of people dispensed at least 1000 mg prednisolone‐equivalent during 2018, stratified by inhaler controller dose and use.

**Results:**

124 011 people had been dispensed at least two prescriptions of ICS during 2014–2018 and met the study definition for asthma, of whom 64 112 (51.7%) had also been dispensed OCS, including 34 580 (27.9% of the asthma group) cumulatively dispensed 1000 mg prednisolone‐equivalent or more. Of 138 073 people dispensed OCS at this level, 68 077 (49%) were patients with airway diseases. Dispensing of diabetes and osteoporosis medications was more common for people cumulatively dispensed 1000 mg prednisolone‐equivalent or more. During 2018, 4633 people with asthma using high dose ICS controllers were dispensed 1000 mg prednisolone‐equivalent or more, for 2316 of whom (50%) controller use was inadequate.

**Conclusions:**

Cumulative exposure to OCS in Australia reaches levels associated with toxicity in one‐quarter of patients with asthma using ICS. Cumulative dispensing of potentially toxic OCS amounts often accompanies inadequate inhaler controller dispensing. Better approaches are needed to improve adherence to controller therapy, improve outcomes for people with asthma, and to minimise the use and toxicity of OCS.

**The known:** Oral corticosteroids are used for treating acute asthma attacks, and sometimes for longer term control, but the risk of long term toxicity increases with cumulative doses exceeding 1000 mg prednisolone.

**The new:** One‐quarter of people with asthma who use inhaler controllers are dispensed potentially toxic cumulative oral corticosteroid doses. Controller medication use by about one‐half of those using high dose combination controllers and exposed to high levels of prednisolone is inadequate.

**The implications:** We need better approaches to improving outcomes for people with asthma that also minimise oral corticosteroid use, including detecting and improving poor use of controller therapy.

One in nine Australians has asthma, which entails risks of severe respiratory attacks and death.^1^ Short courses of oral corticosteroids (OCS) are recommended for treating acute asthma attacks.^2^ For a few patients, long term OCS therapy may be required to protect them from repeated and life‐threatening attacks.

Despite their benefits, OCS have well known adverse effects: they can elicit osteoporosis and fracture, diabetes, cataracts, weight gain, sleep apnoea, renal impairment, pneumonia, myocardial infarction, heart failure, stroke, dyslipidaemia, and depression.^3^, ^4^ However, many clinicians are unaware that toxic effects can develop after relatively low cumulative doses, or do not consider the cumulative burden of repeated treatment with OCS.^4^

The risk of many long term adverse effects increases once lifetime exposure to OCS exceeds 1000 mg prednisolone‐equivalent,^5^, ^6^, ^7^ a potentially toxic level that can be reached after just four short courses of prednisolone. The threshold of concern for OCS use may therefore need to be re‐examined and asthma care pathways re‐evaluated. To understand the magnitude of the risk associated with OCS, we analysed Australian dispensing data to estimate the level of OCS prescribing for people with asthma, with a particular focus on the cumulative dispensing of amounts associated with long term toxicity.

## Methods

In our retrospective cohort study, we examined the cumulative prescribing of OCS for adolescents and adults with asthma who required prescriptions for controller therapy with inhaled corticosteroids (ICS).

### Data source and setting

The Australian Pharmaceutical Benefits Scheme (PBS) is a national program that subsidises medication costs for citizens and permanent residents, and for people from countries with reciprocal health care agreements with Australia.^8^ For subsidised medications, the PBS database includes dispensing data for all community pharmacies and private hospitals in Australia, and for public hospital outpatient and discharge dispensing in all states apart from New South Wales and the Australian Capital Territory. A 10% random sample of the PBS dispensing database is available for analysis, and the sample has been validated by the Australian Bureau of Statistics as being representative of the Australian population.^9^ For this study, we extracted data from the 10% PBS sample for the period 1 January 2014 – 31 December 2018.

The primary objective was to estimate the number and proportion of people dispensed OCS for managing asthma during 2014–2018, and the proportion dispensed a cumulative dose of 1000 mg prednisolone‐equivalent or more over the 5‐year period. Secondary objectives were to estimate the number and proportion of patients dispensed a cumulative dose of 1000 mg prednisolone‐equivalent or more during 2018 (ie, over 12 months), stratified by inhaler controller medication use.

### Study population

For the primary outcome (cumulative OCS dispensing over five years), people were included if they were aged at least 12 years and had been dispensed at least two ICS prescriptions (ICS alone or in combination with long acting β‐agonists [ICS/LABA]) in a single calendar year during 2014–2018.

We defined patients with asthma as those who met the above criteria, but who did not meet our criteria for COPD:

- When authority is required from the PBS to prescribe a medication, the prescriber documents the indication. Prescriber‐submitted diagnostic‐specific authority codes for asthma and COPD were available only for the final five months of the study period (1 August – 31 December 2018).
- For prescriptions without prescriber‐submitted codes, seven sets of criteria for identifying people with asthma and excluding those with COPD were piloted; the algorithm with the best discriminatory value was selected by consensus (Supporting Information, table 1). This benchmarking was based on data for patients identified as having asthma or COPD according to prescriber‐submitted authority codes, and the selected algorithm was applied to prescribing data without prescriber‐submitted codes.

The selected algorithm excluded people receiving any LABA alone (other than salmeterol or formoterol) or in combination with a long acting muscarinic agonist (LAMA), or any LAMA other than tiotropium. Patients over 44 years of age who were dispensed a tiotropium formulation other than Spiriva Respimat (the only formulation approved for asthma) were also excluded.

To estimate the proportions of patients we misclassified as being treated for asthma or COPD, we applied the proportions of people in the benchmarking group incorrectly classified by our algorithm to the rest of the study population.

For the secondary outcomes, the study period was 1 January – 31 December 2018, and the study population included people in the 10% PBS data sample who had been dispensed at least two prescriptions for ICS or ICS/LABA and met the study definition of asthma.

### Exposure

For the primary outcome, exposure was the cumulative amount of prednisolone or prednisone (prednisolone‐equivalent) dispensed over five years. A sensitivity analysis assessed only cumulative OCS exposure after ICS had been dispensed to patients twice during the study period.

### Outcomes

The primary outcomes were the number and proportion of people dispensed OCS for the management of asthma during 2014–2018, and the proportion who were dispensed cumulative doses of 1000 mg prednisolone‐equivalent or more.

We compared the numbers of people aged 12 years or more cumulatively dispensed at least 1000 mg prednisolone‐equivalent for treating asthma with OCS dispensing in the following groups:

- patients in the 10% PBS data sample who were not dispensed inhalers during the five‐year period (people without airway disease);
- patients who were dispensed at least two prescriptions for ICS or ICS/LABA in a single calendar year during the five‐year period, but excluded from the asthma group because they were classified as having COPD;
- patients who were dispensed an inhaled medication during the five‐year period, but not two prescriptions for ICS or ICS/LABA in a single calendar year (intermittent or no controller dispensing).

The dispensing of medications for managing diabetes and osteoporosis were also assessed by cumulative OCS dispensing level.

The secondary outcomes were the number and proportion of patients dispensed cumulative doses of 1000 mg prednisolone‐equivalent or more during 2018 (ie, over 12 months), stratified by inhaler controller medication dose (high dose ICS/LABA formulations *v* others; Supporting Information, table 2) and frequency of use (frequent users: dispensed at least 50% of prescriptions needed for 12 months’ continuous inhaler use *v* infrequent users: < 50%). The sources of OCS prescriptions (general practitioners, respiratory specialists, others) during 2018 were also assessed.

### Ethics approval

The Australian Department of Human Services granted ethics approval for this study (reference, RMS0784).

## Results

### Study population

In the 10% PBS data sample, 124 011 people had been dispensed at least two prescriptions of ICS or ICS/LABA during 2014–2018 and met the study definition of asthma; their mean age was 58.0 years (standard deviation, 19.6 years) and 72 174 were women (58%). In this group, 49 087 people (40%) were classified as being treated for asthma according to prescriber‐provided authority diagnostic codes, and 74 924 (60%) according to our algorithm. We estimated that 23 286 people classified as having asthma may actually have had COPD (18.7% of 124 011), and that 10 594 people with asthma (corresponding to 8.5% of the size of the overall group) may have been incorrectly excluded from our asthma group (Supporting Information, table 3).

### Primary outcome: five‐year oral corticosteroid dispensing

In the asthma group, 64 112 of 124 011 people (51.7%) were dispensed OCS; 34 580 people (27.9%) were cumulatively dispensed 1000 mg prednisolone‐equivalent or more. In a sensitivity analysis of data for cumulative OCS dispensing after ICS was dispensed twice during the study period, 58 161 people (46.9%) were dispensed OCS, including 29 762 (24.0%) cumulatively dispensed 1000 mg prednisolone‐equivalent or more.

During 2014–2018, cumulative doses of 1000 mg prednisolone‐equivalent or more were also dispensed to 69 996 people receiving no inhaler medication, 17 873 people with COPD, and 15 624 people dispensed inhaled respiratory medication but minimal or no controller medication. That is, 49% of people cumulatively dispensed 1000 mg prednisolone‐equivalent or more (68 077 people) were presumptively patients with airway diseases (as evidenced by any inhaler dispensing) (Box 1).

Box 1 People aged 12 years or more in the 10% Pharmaceutical Benefits Scheme dispensing data sample dispensed 1000 mg prednisolone‐equivalent or more during 2014–2018, by inhaler medication use and disease classification*

COPD = chronic obstructive pulmonary disease; ICS = inhaled corticosteroids. * No inhaler use: people without airway disease; asthma: people dispensed at least two prescriptions for ICS or ICS/long acting β‐agonist (LABA) combinations within a single calendar year and did not have COPD; COPD: dispensing as for asthma group, but excluded because of COPD; limited ICS use: dispensed at least one inhaled medication, but not at least two ICS or ICS/LABA prescriptions in a single calendar year.

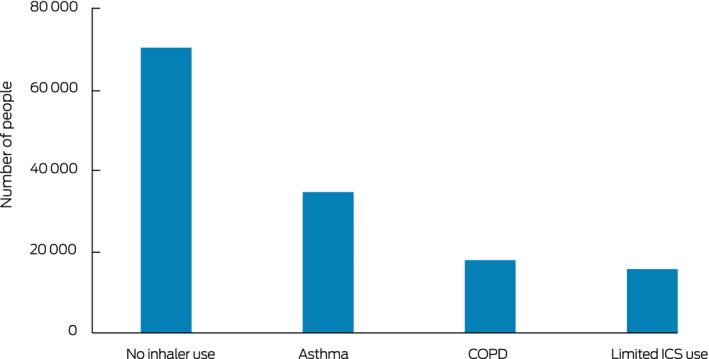

The proportions of people dispensed medications for managing diabetes or osteoporosis were significantly higher for patients cumulatively dispensed 1000 mg prednisolone‐equivalent or more than for those with lower cumulative exposure (Box 2).

Box 2 Dispensing of medications for managing diabetes and osteoporosis to people aged 12 years or more in the 10% Pharmaceutical Benefits Scheme (PBS) dispensing data sample, 2014–2018, by five‐year cumulative prednisolone‐equivalent exposure*

OCS = oral corticosteroids. * All people in the 10% PBS sample dispensed OCS, whether dispensed inhalers on not, with the exception of “No OCS” group (only people dispensed inhalers); absolute numbers are provided in the Supporting Information, table 4. In each panel, > 2500 mg *v* < 1000 mg and 1000–2499 mg *v* < 1000 mg: *P* < 0.001 (two‐tailed test of proportions).

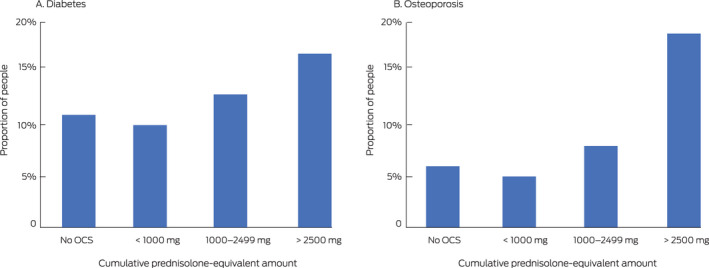

### Secondary outcomes: twelve‐month oral corticosteroid dispensing, by amount and frequency of inhaled corticosteroid use

In the 10% PBS data sample, 65 443 people had been dispensed at least two prescriptions for ICS or ICS/LABA during 2018 and met the study definition of asthma; their mean age was 60.2 years (standard deviation, 18.7 years), and 37 957 were women (58%). In this group, 46 601 people (71%) had also been dispensed high dose ICS/LABA inhalers, of whom 17 600 (38%) were frequent users and 29 001 (62%) infrequent users (Box 3).

Box 3 Dispensing of potentially toxic doses of oral corticosteroids (1000 mg prednisolone‐equivalent or more during 2018) to people aged 12 years or more in the 10% Pharmaceutical Benefits Scheme data sample, by controller medication dose and frequency of use*

ICS/LABA = inhaled corticosteroid/long acting β agonist combination inhaler. * Frequent use: at least 50% of dispensing required for continuous 12‐month use; infrequent use: less than 50% of dispensing required for continuous 12‐month use. Pale wedges: patients cumulatively dispensed 1000 mg prednisolone equivalent or more. Numbers based on 10% sample of the Pharmaceutical Benefits Scheme. ICS/LABA controller formulations defined as high dose: see Supporting Information, table 2.

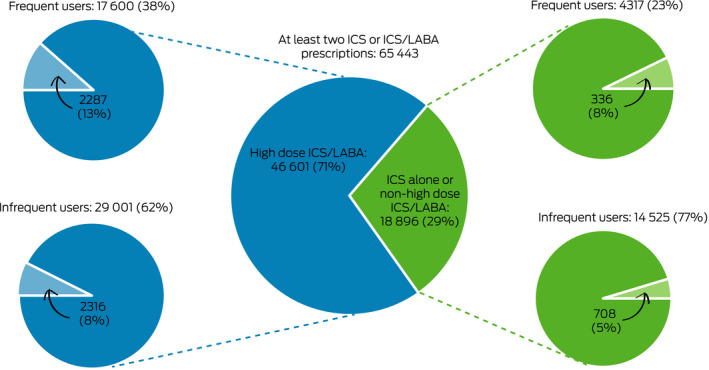

Cumulative dispensing of 1000 mg prednisolone‐equivalent or more during 2018 was recorded for 2287 frequent users (13%) and for 2316 infrequent users of high dose ICS/LABA (8.0%); that is, controller use was inadequate for 50% of those dispensed potentially toxic cumulative OCS doses (2316 of 4603 patients). Cumulative dispensing of 1000 mg prednisolone‐equivalent or more was recorded for 336 of 4317 frequent users (7.8%) and for 708 of 14 525 infrequent users of ICS alone or non‐high dose ICS/LABA (4.9%); that is, controller use was inadequate for 68% of those dispensed potentially toxic cumulative OCS amounts (708 of 1044 patients) (Box 3).

The major prescribers of OCS during 2018 were general practitioners (49 540, 76%), non‐specialists (12 303, 19%), and respiratory specialists (3599, 5.5%).

## Discussion

We have estimated OCS dispensing for the management of asthma in Australia. For 28% of people with asthma using ICS, cumulative dispensing of OCS reached levels associated with long term toxicity. Further, cumulatively high OCS doses were often dispensed to patients whose use of controller medication appears to have been inadequate, suggesting that their need for OCS could be reduced by improving adherence to ICS therapy. Overall, better approaches are needed to improve asthma outcomes whilst minimising OCS use and toxicity.

We estimate that almost 350 000 people with asthma were cumulatively dispensed 1000 mg oral prednisolone‐equivalent or more during 2014–2018, a level associated with long term systemic toxicity.^5^ While we did not specifically examine harms, we found that larger proportion of patients cumulatively dispensed 1000 mg prednisolone‐equivalent or more were also dispensed medications for treating diabetes or osteoporosis than of people receiving lower amounts.

Of all patients in the PBS 10% data sample cumulatively dispensed at least 1000 mg prednisolone‐equivalent over five years, 49% were people using inhaler medication and therefore likely to have had airways disease, including asthma and COPD. This finding is congruent with overseas reports that respiratory disease is the most frequent indication for OCS therapy.^10^

In 2018, potentially toxic range cumulative OCS doses were dispensed to an estimated 22 870 patients with asthma who used high dose ICS/LABA controllers frequently (13% of such users). The proportion of infrequent high dose controller users dispensed high OCS doses was smaller (8.0%), but included a similar number of patients (23 160); this figure could be reduced by strategies that improve controller treatment adherence and thereby reduce the need for OCS therapy.

Apart from this major exception, our findings do not address the appropriateness of OCS use in Australia, but other studies have identified major deficiencies in asthma care.^11^ The magnitude of OCS dispensing we report should therefore prompt re‐examination of the adequacy of asthma management. The need for short courses of OCS to treat asthma attacks has always indicated failure of disease control; repeated courses may also lead to OCS toxicity.^12^

Three‐quarters of OCS prescriptions were provided by general practitioners. Patients for whom OCS therapy is needed should undergo a comprehensive asthma review by their general practitioners, focusing on education and medication adherence.^13^ As subjective assessment is highly inaccurate,^14^ methods for objectively checking adherence^15^ and more reliable monitoring of disease activity are needed. Until they are available, patient review should include checking their inhaler and peak flowmeter technique, updating their treatment plans, and assessing them for treatment escalation according to national guidelines^16^ or for specialist review.^17^ People who continue to need OCS while being cared for by specialists should be considered for systematic assessment at asthma centres for difficult‐to‐treat patients.^18^, ^19^, ^20^, ^21^ A subset of patients with a severe asthma phenotype are eligible for targeted therapy with new monoclonal agents that can reduce attack frequency and therefore the need for short course OCS therapy.^22^, ^23^

Patterns of OCS prescribing in the community should be further examined to determine when it reflects inadequacies in management (education, home monitoring, early detection, action plan), when it reflects severe uncontrolled disease, and when it is unnecessary. This will guide efforts to improve the appropriateness of OCS prescribing.

Knowledge translation and education programs could improve prescriber awareness of the risks associated with cumulative OCS dispensing, and national prescribing alert systems would systematically flag cumulative exposure. Finally, the large number of patients using controller medication infrequently who are dispensed OCS highlights the need for effective strategies for detecting and improving poor adherence to medication by patients with asthma.

### Strengths and limitations

The 10% PBS dispensing data sample provided a representative sample of the national population. The nature of the database facilitated estimation of cumulative OCS exposure, which permits comparison with long term OCS toxicity studies on similar time scales.^5^

The PBS database, however, does not include comprehensive diagnostic information. For much of the study population, excluding people with COPD relied upon an indirect approach based on the pattern of medications dispensing. This could be partially mitigated by our using prescriber‐supplied authority codes when available and estimating classification error for patients without authority codes. ICS can be prescribed for indications other than asthma and COPD, such as chronic cough. As the PBS does not collect information on indications for OCS prescribing, they may have been prescribed for diagnoses other than asthma. We did not examine ICS or OCS dispensing prior to 2014, and may therefore have underestimated lifetime exposure; this may have affected our analysis of the prescribing of medications for diabetes and osteoporosis for some patients. Finally, we could not ascertain the proportion of OCS dispensed that was actually taken by patients. However, the association of more frequent dispensing of osteoporosis and diabetes medications to people with higher cumulative exposure to OCS is consistent with linkage with OCS dispensing, use, and toxicity.

### Conclusion

One‐quarter of patients with asthma who use ICS‐containing controllers were dispensed potentially toxic cumulative amounts of OCS, and many patients dispensed OCS are not adhering to appropriate ICS controller therapy. The need for frequent short courses or long term OCS therapy is neither benign nor acceptable. Measures for assuring adequate asthma control should include thorough primary care review and, when appropriate, referral to specialist care or specialised asthma centres for difficult‐to‐treat patients.

## Competing interests

Mark Hew has received grants‐in‐aid, speaker fees, and fees for serving on the advisory boards of GlaxoSmithKline, AstraZeneca, Novartis, Teva, Sanofi and Seqirus, all paid to his institutional employer, Alfred Health. Vanessa McDonald has received research grants from GSK and AstraZeneca for unrelated work, and speaker honoraria from GSK, AstraZeneca, and Menarini for education unrelated to this work. Phil Bardin has received advisory board fees from Boehringer Ingelheim, GSK, AstraZeneca, Sanofi and Novartis, and unrestricted grant funding from GSK. Li Ping Chung has received speaker and consultancy fees and conference expenses from AstraZeneca, Novartis, GSK, Boehringer Ingelheim and Menarini. Claude Farah has received honoraria from Boehringer Ingelheim, AstraZeneca, GSK and Sanofi for attending and speaking at educational meetings and advisory boards. Amanda Barnard has received funding from AstraZeneca for travel to education meetings and advisory groups. Peter Gibson has received speaker fees and grants to his institution from AstraZeneca, GlaxoSmithKline and Novartis for unrelated projects. John Upham has received speaker fees, consultancy fees and conference expenses from AstraZeneca, Novartis, GSK, Boehringer Ingelheim and Sanofi Genzyme, none directly related to this study.

## Supporting information

Supplementary tables
