# Supplementary material for: Cumulative dispensing of high oral corticosteroid doses for treating asthma in Australia
Source: Med J Aust. 2020 Sep 9;213(7):316–20. doi: 10.5694/mja2.50758 (PMC7589219; doi:10.5694/mja2.50758)
Supplement: Supplementary file 1 — Supplementary tables [file MJA2-213-316-s001.pdf]

The Medical Journal of Australia

Supporting Information

Supplementary tables

This appendix was part of the submitted manuscript and has been peer reviewed.
It is posted as supplied by the authors.

Appendix to: Hew M, McDonald VM, Bardin PG, et al. Cumulative dispensing of high oral corticosteroid doses for treating asthma in Australia. *Med J Aust* 2020; doi: 10.5694/mja2.50758.

Table 1. Pilot algorithms for identifying people with asthma and excluding those with chronic obstructive pulmonary disease (COPD), benchmarked according to data for 70 353 people over 12 years of age with prescriber-submitted authority codes for asthma or COPD

			Classification				
			Asthma		COPD		
	Algorithm	Total number	Correct	Incorrect	Correct	Incorrect	Ambiguous*
1	> 44 years: COPD	70 353	13 792 (33.8%)	26 980 (66.2%)	22 198 (81.3%)	5117 (18.7%)	2266
2	> 44 years: COPD; *or*<br>≤ 44 years + any LABA (except salmeterol or formoterol), LAMA/LABA, LAMA (except tiotropium): COPD	70 353	13 221 (32.4%)	27551 (67.6%)	22 638 (83.0%)	4677 (17.0%)	2266
3	Any LABA (except salmeterol or formoterol), LAMA/LABA, LAMA (except Spiriva Respimat): COPD	70 353	34 038 (83.5%)	6734 (16.5%)	11 274 (41.3%)	16 041 (58.7%)	2266
4†	Any LABA (except salmeterol or formoterol), LAMA/LABA, LAMA (except tiotropium): COPD; *or*<br>> 44 years + non-Spiriva Respimat: COPD	70 353	33 831 (83.0%)	6941 (17.0%)	12 059 (44.1%)	15 256 (55.9%)	2266
5	Non-tiotropium LAMA: COPD	70 353	38 815 (95.2%)	1957 (4.8%)	2916 (10.7%)	24 399 (89.3%)	2266
6	> 44 years + tiotropium: COPD	70 353	33 207 (81.4%)	7565 (18.6%)	11 257 (41.2%)	16 058 (58.8%)	2266
7	Any LABA (except salmeterol or formoterol), LAMA/LABA, LAMA (except tiotropium): COPD	70 353	37 496 (92.0%)	3276 (8.0%)	8566 (31.4)	18 749 (68.6%)	2266

LABA = long acting β-agonist; LAMA = long acting muscarinic antagonist.

* Patients dispensed medication on prescriber-submitted authority codes for both asthma and COPD. during the time period.

† Selected to exclude people with COPD without prescriber-supplied authority codes.

Table 2. Inhaled corticosteroid/long acting β-agonist fixed dose combination inhalers defined as high dose for analysis reported in Box 4

Inhaler, dose (μg)	Components
Seretide Accuhaler 250/50	Fluticasone + salmeterol
Seretide Accuhaler 500/50	Fluticasone + salmeterol
Flutiform 250/10	Fluticasone + eformoterol
Symbicort Rapihaler 200/6	Budesonide + eformoterol
Symbicort Turbuhaler 200/6	Budesonide + eformoterol
Symbicort Turbuhaler 400/12	Budesonide + eformoterol
Breo Ellipta 200/25	Fluticasone + vilanterol
DuoResp Spiromax 400/12	Budesonide + formoterol

Table 3. Benchmarking exercise for estimating classification error for 124 011 people classified as having asthma, 2014–2018

For people with prescriber-provided diagnostic-specific authority codes:

- The authority codes for 1 August – 31 December 2018, allow 100% accurate classification of 49 087 people with asthma.
- Compared with this classification, classification by the chosen algorithm (Table 1, algorithm 4) missed 6941 people with asthma (corresponding to 14.1% of the group) and classified 15 256 people as having asthma (31.1% of 49 087) who did not have asthma according to authority coding.

For the overall asthma group:

- Of 124 011 people included, 49 087 were classified as having asthma according to prescriber-provided authority codes; 74 924 were classified with the algorithm.

For the group of 74 924 people classified with the algorithm:

- Estimated number of people with asthma missed: 14.1% of 74 924 = 10 594 people.
- People with chronic obstructive pulmonary disease (COPD) and other non-asthma conditions included in asthma group: 31.1% of 74 924 = 23 286 people.

Estimated classification error in the overall asthma group:

- Proportion of asthma patients missed is 10 594, corresponding to 8.5% of overall group.
- Proportion of included people with COPD and other non-asthma conditions: 23 286/124 011 = 18.7%.

Table 4. Dispensing of medications for managing diabetes and osteoporosis to people aged 12 years or more in the 10% Pharmaceutical Benefits Scheme dispensing data sample, 2014–2018, by five-year cumulative prednisolone-equivalent exposure* (data for Box 2 in the main text)

	No OCS	1–999 mg	1000–2499 mg	≥ 2500 mg	Total
Diabetes medications					
Numerator	20 958	24 161	11 354	9332	65 805
Denominator	188 203	246 030	89 981	53 911	578 125
Proportion	11.1%	9.8%	12.6%	17.3%	11.4%
Osteoporosis medications					
Numerator	10 738	11 609	6820	10 394	39 561
Denominator	188 203	246 030	89 981	53 911	578 125
Proportion	5.7%	4.7%	7.6%	19.3%	6.8%

* All people in the 10% PBS sample dispensed OCS, whether dispensed inhalers on not, with the exception of “No OCS” group (only people dispensed inhalers). In each panel, > 2500 mg *v* < 1000 mg and 1000–2499 mg *v* < 1000 mg: *P* < 0.001 (two-tailed test of proportions).
